# The Link Between Inflammaging and Degenerative Joint Diseases

**DOI:** 10.3390/ijms20030614

**Published:** 2019-01-31

**Authors:** Elena Rezuș, Anca Cardoneanu, Alexandra Burlui, Andrei Luca, Cătălin Codreanu, Bogdan Ionel Tamba, Gabriela-Dumitrița Stanciu, Nicoleta Dima, Codruța Bădescu, Ciprian Rezuș

**Affiliations:** 1 Department of Rheumatology and Physiotherapy, “Grigore T. Popa’’ University of Medicine and Pharmacy Iași, University Street no. 16, 700115 Iași, Romania; elena.rezus@umfiasi.ro (E.R.); anca.cardoneanu@umfiasi.ro (A.C.); maria-alexandra.burlui@umfiasi.ro (A.B.); 2 Advanced Center for Research and Development in Experimental Medicine (CEMEX), “Grigore T. Popa’’ University of Medicine and Pharmacy Iași, Mihail Kogălniceanu Street no. 9-13, 700454 Iasi, Romania; gabriela-dumitrita.s@umfiasi.ro; 3 Center for Rheumatic Diseases, “Carol Davila” University of Medicine and Pharmacy Bucharest, Eroii Sanitari no.8 Boulevard, 050474 Bucharest, Romania; catalin.codreanu@reumatologiedrstoia.ro; 4 Department of Internal Medicine, “Grigore T. Popa’’ University of Medicine and Pharmacy Iași, University Street no. 16, zip code 700115 Iași, Romania; nicoleta.dima@umfiasi.ro (N.D.); minerva.badescu@umfiasi.ro (C.B.); ciprian.rezus@umfiasi.ro (C.R.)

**Keywords:** inflammaging, osteoarthritis, chondrosenescence

## Abstract

Aging is an inevitable process in the human body that is associated with a multitude of systemic and localized changes. All these conditions have a common pathogenic mechanism characterized by the presence of a low-grade proinflammatory status. Inflammaging refers to all the processes that contribute to the occurrence of various diseases associated with aging such as frailty, atherosclerosis, Alzheimer’s disease, sarcopenia, type 2 diabetes, or osteoarthritis. Inflammaging is systemic, chronic, and asymptomatic. Osteoarthritis and many age-related degenerative joint diseases are correlated with aging mechanisms such as the presence of an inflammatory microenvironment and the impaired link between inflammasomes and autophagy. There is a close relationship between chondrocyte activity and local articular environment changes due to cell senescence, followed by secretion of inflammatory mediators. In addition, systemic inflammaging can lead to cartilage destruction, pain, disability, and an impaired quality of life. The purpose of this review is to summarize the main mechanisms implicated in inflammaging and the connection it has with degenerative joint diseases.

## 1. Introduction

**Figure 1:**
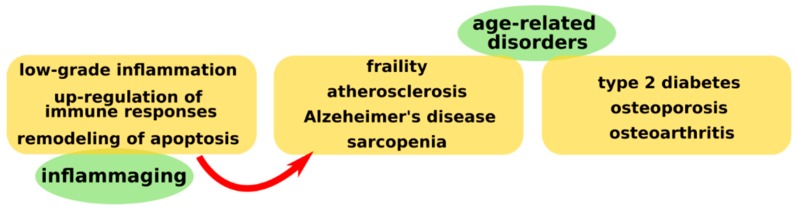
This figure depicts the main mechanisms implicated in inflammaging, as well as the main associated diseases with this process. Inflammation is characterized by the presence of systemic low-level inflammation due to the excess secretion of cytokines with a proinflammatory role. Along with these, the aging of the body also presents an imbalance of the immune system that leads to up-regulation of immune responses. Older age also shows a decrease in apoptotic processes. All of these mechanisms seem to be incriminated in the pathology of age-related disorders such as accelerated atherosclerosis, constitutional sarcopenia and frailty, type 2 diabetes, or rheumatic diseases such as arthrosis or osteoporosis.

It is well known that the aging process is associated with the appearance of various pathologies such as frailty, atherosclerosis, Alzheimer’s disease, sarcopenia, type 2 diabetes, or osteoporosis [1,2]. All these conditions have a common pathogenic mechanism characterized by the presence of a low-grade proinflammatory status (Figure 1).

The term “inflammaging” was first used in 2000 by Franceschi [3] and refers to all the processes that contribute to the occurrence of various diseases associated with aging. Inflammaging represents a low-grade inflammatory status and together with the up-regulation of the immune response, as well as with the remodeling of apoptosis, contributes to these age-related disorders [3]. Inflammaging is systemic, chronic, and asymptomatic. Osteoarthritis and many age-related degenerative joint diseases are correlated with aging mechanisms such as the presence of an inflammatory microenvironment and the impaired link between inflammasomes and autophagy [4].

## 2. The Link between Aging and Articular Cartilage

Articular cartilage is a thin connective tissue that covers the surfaces of the joints. Cartilage is composed of specialized cells called chondrocytes that produce a large amount of collagenous extracellular matrix, rich in proteoglycan and elastin fibers. Chondrocytes derive from chondroblasts that are trapped in lacunae and mature in chondrocytes. Chondrocyte metabolism responds to both mechanical (mechanical load, hydrostatic pressure changes) and chemical stimuli (growth factors, cytokines). Because of the lack of blood vessels and progenitor stem cells, the capacity of self-repair of the articular cartilage is limited [5].

A recently published study has highlighted the changes in articular cartilage in the situation of in vitro monolayer culture. Significant changes in cell phenotype have been observed. Cells modification of the normal shape with a flattened one, altered secretory capacity and synthesis of collagen type X has been noted. Furthermore, a decrease in specific secretion products such as glycoproteins, proteoglycans, or type II collagen was highlighted. All of these changes have been attributed to the “stress responses” induced by cultivation conditions [6].

**Figure 2:**
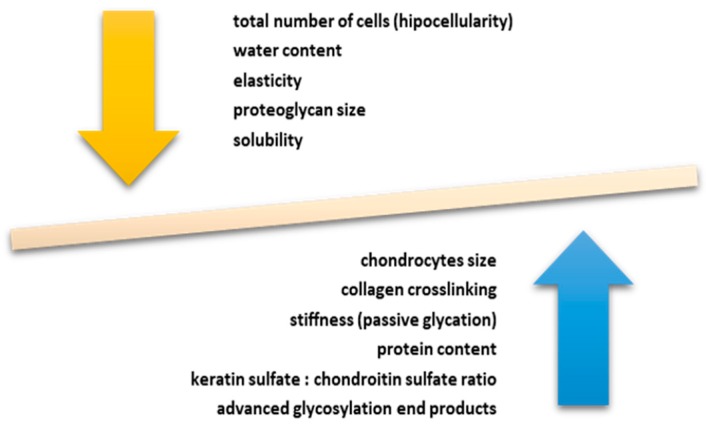
Main changes in articular cartilage due to aging process. Aging is responsible for the senescence of chondrocytes and for the specific modifications that appear in the structure of the cartilage. The anabolic processes are slowed down, and the catabolic ones accelerated. Significant changes in cell phenotype have been observed. Cells modification of the normal shape with a flattened one, altered secretory capacity and synthesis of collagen type X has been noted. A decrease in specific secretion products, such as glycoproteins, proteoglycans or type II collagen, was also highlighted. The aging of articular cartilage is characterized by a decrease in cellularity, dehydration, decreased elasticity and solubility, and decreased proteoglycan molecule sizes. On the other hand, an increase in chondrocyte size, cartilage stiffness, protein content and glycosylation products were observed.

Aging is responsible for the senescence of chondrocytes and for the specific modifications that appear in the structure of the cartilage [7] with the main changes being listed in (Figure 2).

As we know, the incidence of osteoarthritis (OA) increases proportionally with age, but we can’t consider it a direct consequence of aging [8]. The term “chondrosenescence” refers to all “age-dependent deterioration of chondrocytes as a consequence of replicative (intrinsic) and stress-induced [extrinsic] factors” [9]. There is a strong correlation between inflammaging, the presence of inflammasomes, autophagy, and chondrosenescence (Figure 2) [9,10].

The main changes at the articular cartilage level due to the aging process are represented in Figure 2, with respect to the modifications over chondrocytes, collagen, proteins, and keratin sulfate/chondroitin sulfate ratio together with changes in cellularity, elasticity, solubility, and the presence of dehydration.

**Figure 3:**
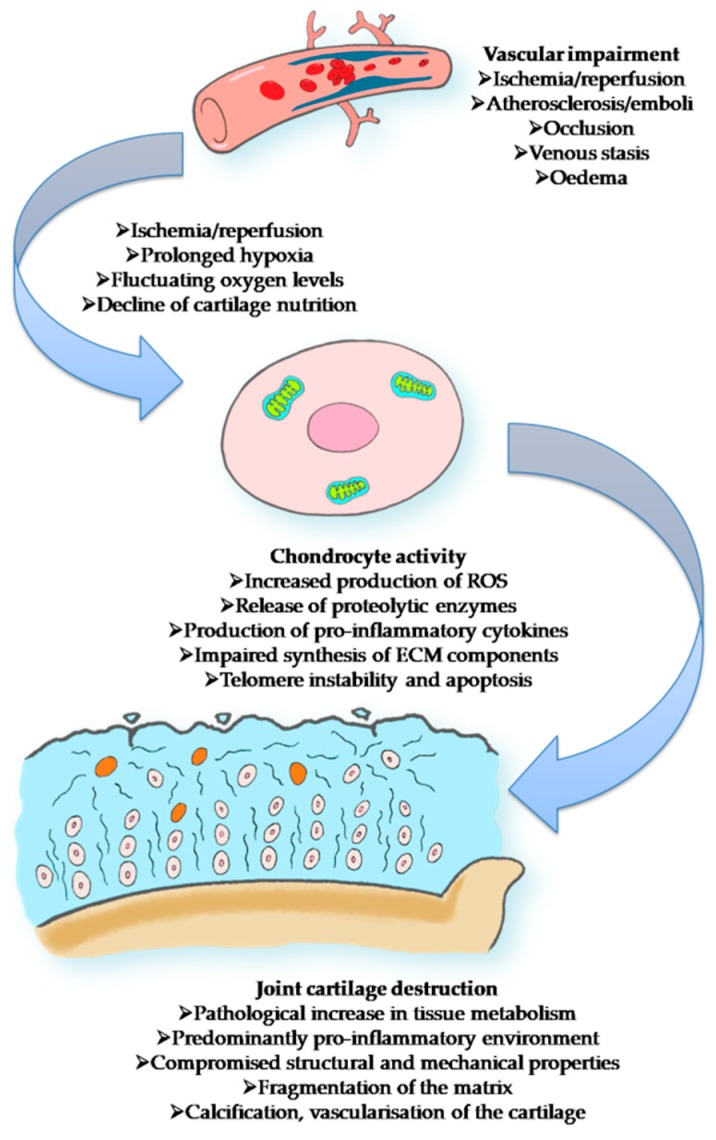
Vascular impairment leading to a disruption in chondrocyte activity and the subsequent destruction of joint cartilage. It has been shown that decreased blood flow results in poor nutrition, as well as the disruption of chondrocyte function and fluctuating oxygen levels promoting a pathological augmentation in metabolic activity [11,12]. In addition, in cases of prolonged hypoxia, chondrocytes release high amounts of proinflammatory cytokines and reactive oxygen species (ROS), which contribute to the development of a proinflammatory microenvironment [13]. Chondrocyte telomere instability as well as apoptosis may also be bolstered by the presence of ROS [14]. Moreover, oxidative stress induces a reduction of extracellular matrix (ECM) components by chondrocytes, leading to an alteration of cartilage structure and the subsequent decline of the tissue’s mechanical properties, with the appearance of fissures and fragmentation [15].

Moreover, the cartilage suffers from changes in blood flow and, secondarily to this, from the modifications in chondrocyte activity, overall leading to joint cartilage destruction (Figure 3).

## 3. Mechanism of Inflammaging and Implications in OA

**Figure 4:**
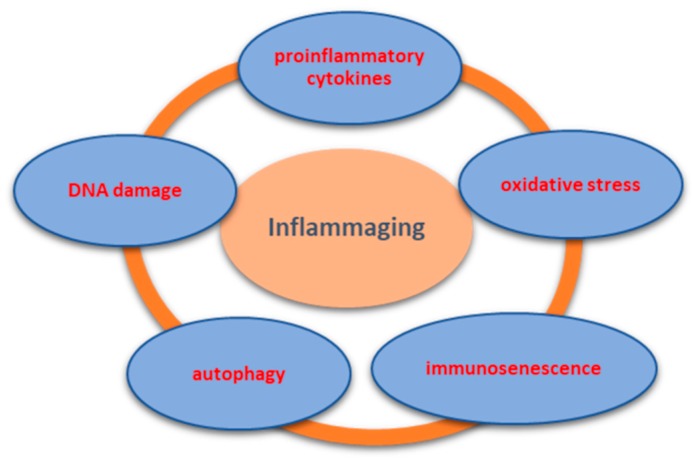
The main multifactorial mechanisms related to inflammaging. The aging process of the body is complex, influencing numerous cellular, immunological, and even genetic mechanisms. Thus, a proinflammatory status characterized by an excess secretion of proinflammatory cytokines such as interleukins (-1, -4, -6, -15), alpha tumor necrosis factor, or gamma interferon was revealed. Along with this inflammatory phenotype, an increase in the oxidative stress has been highlighted, which results in the accumulation of oxygen metabolites. Furthermore, in the aging process, there is a decrease in the autophagy capacity, which determines pronounced proinflammatory responses and mitochondrial damage. DNA damage response is directly related to telomere shortening and favors proinflammatory status through its action on stem cells, fibroblasts, or macrophages, thus exacerbating the phenomenon of inflammaging.

The mechanisms of inflammaging are not fully understood. However, current data supports its multifactorial etiology, including increased number of proinflammatory cytokines, oxidative stress, immunosenescence, autophagy, or cellular DNA damage, further detailed in Figure 4 [1,3,4].

Ageing has consequences that lead to changes in the immune system, referred to as immunosenescence, and attributed to evolution on the one hand but also related to a chronic antigenic load that promotes pathologies with an inflammatory common ground, as recent theories describe. 

Systemic low-grade inflammation is the main pathogenic factor for chronic disorders related to aging, including OA [16,17]. Data from the literature highlighted increased levels of IL-6 and C-reactive protein (CRP)in patients with knee OA and correlated them with the progression of the disease [18,19]. Other studies demonstrated a relationship between the levels of proinflammatory cytokines and physical symptoms such as articular functionality or the level of the pain [20,21,22,23]. In older cases with knee OA, physical mobility decreased directly proportional to the increase of soluble receptors for TNFα [24]. Furthermore, elevated levels of CRP and TNFα have been associated with higher pain levels in patients with knee arthritis [25]. 

Research conducted on cultures of articular chondrocytes from older patients highlighted increased levels of IL-7 [26] and IL-1β [27] compared to young adults or age-matched normal controls. 

Senescent cells from the articular cartilage have the ability to develop a senescence-associated secretory phenotype, which includes growth factors, chemokine, matrix metalloproteinase (MMP), and a lot of inflammatory cytokines [28]. This inflammatory environment is conducive to developing OA, being found in affected cartilage or in the synovial fluid [29]. 

**Figure 5:**
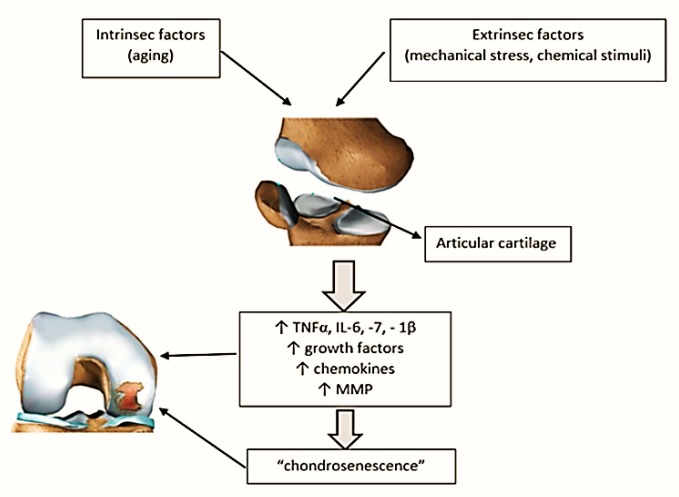
Mechanisms of osteoarthritis in aging. The incidence of osteoarthritis increases proportionally with age, but it cannot be considered a direct consequence of aging. The term “chondrosenescence” refers to all “age-dependent deterioration of chondrocytes as a consequence of replicative (intrinsic) and stress-induced (extrinsic) factors”. Intrinsic factors refer to the aging process, while extrinsic factors include physical-mechanical factors or different chemical stimuli. All these factors act on articular cartilage, making it vulnerable. As a consequence, an inflammatory environment characterized by increased proinflammatory cytokines (TNFα, IL-6,-7,-1β), chemokine, and activated proteinase occurs locally. All these lead to the aging process of chondrocytes (chondrosenescence), which favors the appearance of degenerative joint modifications.

Intrinsic factors in the aging process in association with extrinsic factors such as mechanical overload or different chemical stimuli act on articular cartilage. As a consequence, an inflammatory environment characterized by increased proinflammatory cytokines, chemokine, and activated proteinase occurs locally. All these lead to the aging process of chondrocytes (chondrosenescence), which favors the appearance of degenerative joint modifications (Figure 5).

Senescent cells have a particular phenotype characterized by the formation of senescence-associated heterochromatin, by high expression of cyclin-dependent kinase inhibitor (p16INK4a), and senescence-associated β-galactosidase [30,31]. It has also beendemonstrated that senescence-associated β-galactosidase is only present in OA samples compared with normal cartilage from older patients having hip fractures [32]. An increased level of cyclin-dependent kinase inhibitor, together with high DNA damage and telomere length reduction, were found in aging chondrocytes from OA cartilage [33]. 

One study highlighted that telomere length reduction in peripheral leucocytes was correlated with radiological signs of hand OA [34]. In addition, it seems that free oxygen radicals and oxidative stress can cause DNA damage and shortening of telomeres [35]. 

Data published by Forsyth et al. demonstrated increased secretion of MMP-13 in chondrocytes from older adults compared to young adults [28]. MMP-13 is a major mediator regarding the cleavage of type II collagen. Thus, in the urine of patients over 50 years old, high levels of degradation products of type II collagen have been found [36].

Besides all these data, aging of chondrocytes is also characterized by a decrease in anabolic and proliferative response to cell growth factors such as transforming growth factor-beta (TGF-β), platelet derived growth factor (PDGF), or insulin-like growth factor-I (IGF-I) [37]. In addition, at the level of aging chondrocytes, the synthesis of proteoglycans determined by morphogenic protein-6 (BMP-6) is decreased [38].

The main changes occurring at the level of aged chondrocytes that are directly involved in the process of degradation of articular cartilage and in the onset of OA are presented in Table 1.

Specialized studies speak of the existence of a medical interdisciplinary specialty called “geroscience” that examines the link between biological processes in aging and various diseases such as OA [40]. In OA, the primary role is attributed to local or systemic low-grade systemic inflammation [26,41,42]. Along with low-grade inflammation, other mechanisms of aging like mitochondrial dysfunction, genomic instability, telomere shortening, oxidative stress, autophagy, cellular senescence, or altered intercellular communications have been proposed to be an integral part of the pathophysiology of aging-related diseases [43].

### 3.1. ProInflammatory Cytokines

**Figure 6:**
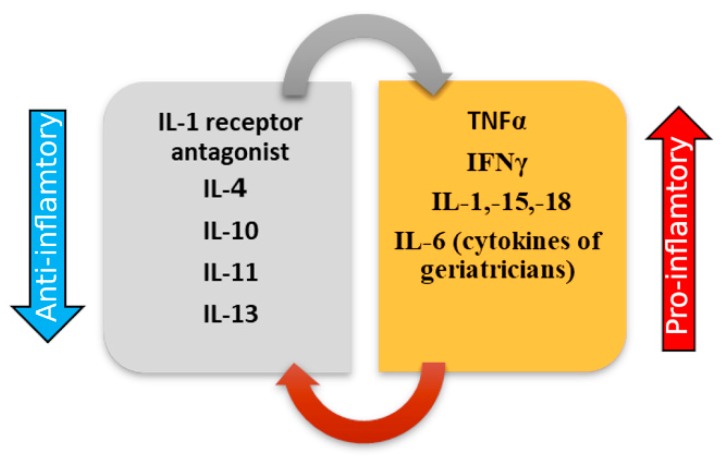
Modifications related to anti-inflammatory and proinflammatory cytokines in the process of inflammaging. Aging is associated with the presence of a low-grade proinflammatory status that maintains all other pathogenic processes. Thus, studies have shown that the most important proinflammatory cytokines involved in the process of inflammaging are tumor necrosis factor α (TNFα), interferon γ (IFNγ), and interleukins (IL)—IL-1, IL-6, IL, IL-15, and IL-18 [5]. IL-6 can be considered a predictive marker of inflammaging, being called the cytokine of geriatricians. The polymorphisms in the promoter C/G 174 on the IL-6 gene is related to immune-inflammatory responses and affects serum IL-6 concentrations.

Low-grade inflammatory status refers to an imbalance between pro- and anti-inflammatory cytokines (Figure 6). The most important proinflammatory cytokines involved in the process of inflammaging are tumor necrosis factor α (TNFα), interferon γ (IFNγ), and interleukins (IL)—IL-1, IL-6, IL-15, IL-18, respectively [44]. These molecules can have pleiotropic effects, stimulating immune reactions. 

The most important cytokine in age-related disorders seems to be IL-6, being associated with chronic morbidity, mortality, and disability [45,46]. Furthermore, studies proved that IL-6 can be considered a predictive marker of inflammaging [46], being called the cytokine of geriatricians [47]. 

High levels of proinflammatory cytokines including IL-6, IL-1, and TNFα have a crucial role in the aging process by creating an inflammatory environment in most of the organs and body tissues [48]. A study that included old horses highlighted increased levels of TNFα, IL-15, IL-18, and IL-1β in peripheral blood samples [49]. 

The fragility, due to the overproduction of proinflammatory cytokines, associated with physical inactivity, hormonal changes, and diet deficiencies, causes a favorable environment for the appearance of osteopenia and sarcopenia [50].

Data from the literature sustain the role of genetical changes in this susceptibility to inflammaging. The polymorphisms in the promoter C/G 174 on the IL-6 gene is related to immune-inflammatory responses and affect serum IL-6 concentrations [51]. Furthermore, the polymorphism of toll-like receptor 4 and of IL-10 can influence the inflammatory mechanisms [52]. The association of low levels of IL-10 with increased levels of IL-6 can improve the ability to fight pathogens [53].

Although it is still unclear, it seems that the shortening process of the telomeres is affected and associated with systemic inflammation in older people. A study which included 1962 participants demonstrated that a shorter telomere length was correlated with an increased level of TNF α and IL-6 [54]. Other studies however, found that the correlation between shorter telomeres and increased levels of IL-6 becomes non-significant when other factors such as age, gender, diet, or income were included in the analysis, or that high levels of CRP were not accompanied necessarily by shortening of the telomeres [55,56].

**Figure 7:**
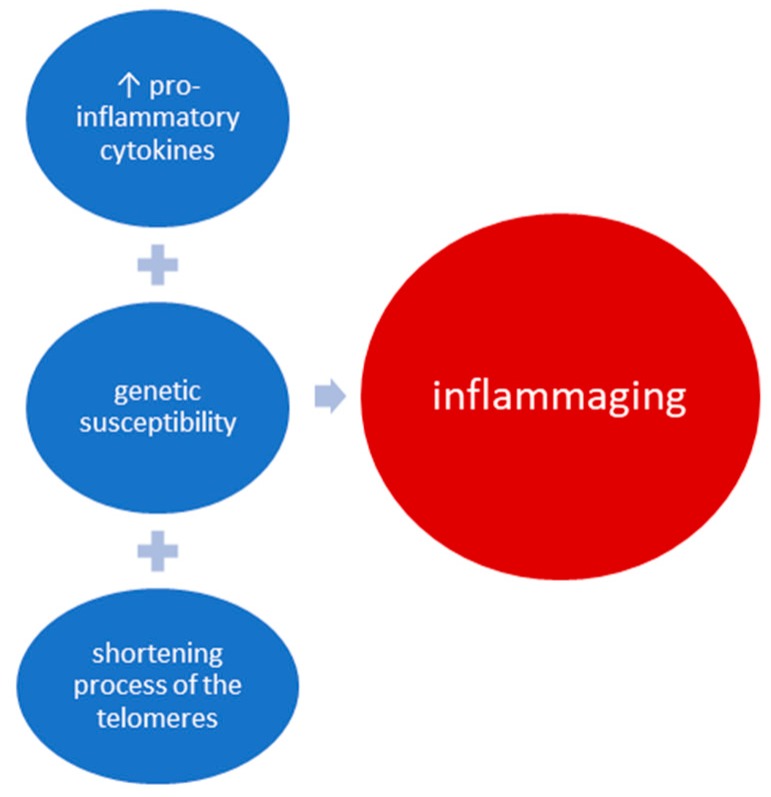
The link between proinflammatory cytokines, genetic susceptibility, and DNA changes that occurs with inflammaging. The etiology of immunosenescence includes genetic, environmental, and immune factors. The damage of innate immunity refers to monocytes, neutrophils, natural killer, and dendritic cells and is characterized by the reduction of phagocytosis and superoxide production. The damage of acquired immunity includes B and T lymphocytes and determines thymus atrophy, increased proinflammatory cytokines, and autoreactivity. High levels of proinflammatory cytokines including IL-6, IL-1, and TNFα have a crucial role in the aging process by creating an inflammatory environment in most of the organs and body tissues. Systemic low-grade inflammation can determine stem cell aging through the activation of the signaling pathways (NF-kB, TOR, JAK/STAT). An important role in the process of replicative senescence and age-related diseases belongs to DNA damage response, which is directly related to telomere shortening. DNA damage response favors proinflammatory status through its action on stem cells, fibroblasts, or macrophages, thus exacerbating the phenomenon of inflammaging.

These three processes are the main mechanisms involved in the pathogenesis of the aging process characterized by a low-grade systemic inflammation. Thus, we can support the view that proinflammatory status characterized by a high titer of proinflammatory cytokines, along with genetic susceptibility, corroborated with the shortening process of the telomeres, lead to the process of inflammaging (Figure 7).

Both the progression and debut of OA seems to be linked to cytokines. In the synovial fluid proinflammatory cytokines accumulate together with anti-inflammatory ones forming a combination entitled cytokine network that can modulate the process of degradation of the cartilage matrix [57,58].

Recent publications, especially proteomic studies of synovial fluid, have identified interleukin-17 [59] to be associated with OA development. Moreover, interleukin-18 and metabolomics profiling indicates that OA could be divided into different subgroups metabolically [60]. Similarly, interleukin-37 also known as IL 1F7, is associated with OA disease activity being correlated with the suppression of proinflammatory cytokine production such as IL-1β, TNF-α, and IL-6, at the synovial cell level [61]. Possible novel therapeutic targets could be discovered if we take into account other inflammatory, anti-inflammatory, and modulatory cytokines associated with OA rather than the presence of IL-1 and TNF-α, which often show poor correlation with the osteoarthritic joint [58].

### 3.2. Oxidative Stress

Oxidative damage through the accumulation of reactive oxygen species (ROS) leads to what is believed to be a remodeling of the immune system to which the body tries to adapt, but in failing to do so, as is the case of elderly patients, predisposition to chronic inflammatory conditions appears [3].

Data sustain the link between oxidative stress, inflammaging, and immunosenescence [62,63]. Mitochondria are considered to be a source of oxygen metabolites during oxidative phosphorylation. The accumulation of the metabolites of oxygen can determine the damage of nucleic acids, proteins, or lipid membranes, inducing apoptotic mechanisms and deoxyribonucleic acid (DNA) damage, especially increasing the risk of cancer [47,64]. 

There is a relation between the function of immune cells and the redox state. High levels of antioxidants can decrease the oxidative stress and slow down the aging process [65,66]. Directly related to this, the literature speaks about the oxidation-inflammatory theory of aging [67]. 

The accumulation of oxygen metabolites could accelerate the process of cellular aging and increase apoptosis by decreasing the adenosine triphosphate (ATP) levels and increasing the porosity of the cellular membranes [68]. 

Aging of the body is associated with an increase in the oxidative phosphorylation process, which results in the accumulation of oxygen metabolites. Reactive oxygen species include peroxides, superoxide, hydroxyl radical, singlet oxygen, and alpha-oxygen. All of these have important roles in cell signaling and homeostasis processes. The most important harmful effects of reactive oxygen species on the cell are damage of DNA or RNA, oxidations of polyunsaturated fatty acids in lipids (lipid peroxidation), oxidations of amino acids in proteins, and oxidative deactivation of specific enzymes by oxidation of co-factors.

**Figure 8:**
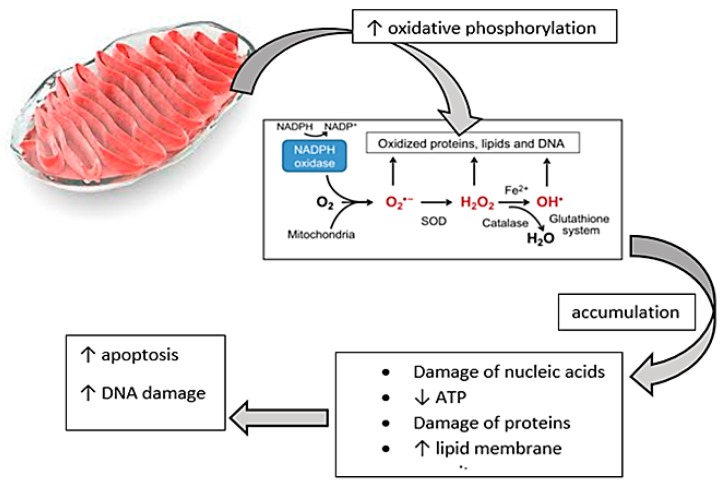
Representation of mitochondrial changes due to increased oxidative phosphorylation with respect to DNA modification and apoptosis linked to the theory of aging. Mitochondrial dysfunction plays an essential role in the appearance of low-level systemic inflammation that characterizes aging processes. Aging of the body is associated with an increase in the oxidative phosphorylation process, which results in the accumulation of oxygen metabolites. Reactive oxygen species include peroxides, superoxide, hydroxyl radical, singlet oxygen, and alpha-oxygen. All of these have important roles in cell signaling and homeostasis processes. The most important harmful effects of reactive oxygen species on the cell are damage of DNA or RNA, oxidations of polyunsaturated fatty acids in lipids (lipid peroxidation), oxidations of amino acids in proteins, and oxidative deactivation of specific enzymes by oxidation of co-factors. All these structural and genomic changes cause an increase in apoptotic processes and affect genetic transcription, leading to shortening of telomeres.

Oxidative stress is also responsible for accelerated apoptosis and cellular damage, which in turn leads to the emergence of various pathologies associated with aging (Figure 8). 

Due to the avascular characteristic of articular cartilage, synovial fluid, and subchondral bone provide oxygen, nutrients, and antioxidants to chondrocytes by diffusion [44,69]. Studies proved that anaerobic conditions occurring in vascular diseases are the first step in the development of osteoarthritis [70]. In the case of prolonged hypoxia, chondrocytes secrete elevated levels of reactive oxygen species such as nitric oxide, hydrogen peroxide, or peroxynitrite [11,71]. Experimental studies revealed high levels of nitric oxide in OA cartilage, which has the property to stop extracellular matrix formation [72]. 

Nitric oxide (NO) is synthesized by different NO synthases (NOS) through the oxidation of the guanidino nitrogen of l-arginine. Endothelial, mitochondrial, and neural NOs are inducible and have a positive role in metabolic processes [73]. It seems that inducible NOS (iNOS) have an important role in OA because their inhibition is associated with stopping the loss of glycosaminoglycans in joint diseases [74,75]. An important role in the pathogenesis of OA has been assigned to neuronal NOS whose activity has been shown to be elevated in chondrocytes [76]. 

A recently published study analyzed the effect of inhibition of selective inducible nitric oxide synthase and neuronal nitric oxide synthase inhibitors on cartilage regeneration. The study included 27 Wistar rats which had saline solution, neuronal nitric oxide synthase inhibitor 7-nitroindazole, inducible nitric oxide synthase inhibitor aminoguanidine, or nitric oxide precursor l-arginine) injected into the knee. After 21 days, the histopathological examination confirmed the positive effect of selective neuronal nitric oxide synthase inhibition on the regeneration of cartilage [77].

Another recent review supports the importance of iNOS in OA development [78]. The authors also made a classification of iNOS inhibitors according to the mechanism of action as follows: structures that interact with calmodulin/flavine cofactors, inhibitors interacting directly with the heme, inhibitors of arginine-binding sites, and drugs that mimic tetrahydrobiopterin cofactor [78,79].

In OA cases, oxidative stress is not limited to destroying articular cartilage and chondrocytes but also affects the extracellular matrix, especially proteoglycans and collagen fibers. Oxygen reactants can cause structural changes in proteins, can modify their activity, and favor the accumulation of cellular debris, thus inducing and maintaining inflammation [11,72]. Oxidative stress can modulate catabolic and anabolic signaling pathways, leading to overproduction of MMP and inflammatory cytokines, and, on the other hand, to the decline in production of extracellular matrix and growth factor expression [80]. 

Oxidative stress plays an important role in the development of degenerative joint changes, especially in prolonged hypoxia conditions. Reactive oxygen species act both directly on chondrocytes, favoring cell death or hyperproduction of inflammatory factors, as well as on the extracellular matrix, inhibiting its production and accentuating the destruction. The process of osteoarthritis is characterized by the increase of catabolic processes followed by the decrease of the anabolic ones (Table 2).

### 3.3. Immunosenescence and DNA Damage 

The definition of cellular senescence refers to the mechanism that leads to an irreversible loss of the proliferation of somatic cells [81]. Furthermore, experimental studies demonstrated that senescent cells, though a precise pathway which involves the release of certain mediators and the stop of proliferative activity, can determine their clearing and tissue regeneration [82,83]. This process is affected in old tissues, leading to the accumulation of these senescent cells [84]. 

Senescent cells have an important role in aging through the secretion of matrix-degrading proteins and proinflammatory cytokines, which is called “senescence-associated secretory phenotype” [85]. 

The etiology of immunosenescence includes genetic, environmental, and immune factors. The damage of innate immunity refers to monocytes, neutrophils, and natural killer and dendritic cells and is characterized by the reduction of phagocytosis and superoxide production. The damage of acquired immunity includes B and T lymphocytes and determines thymus atrophy, increased proinflammatory cytokines, and autoreactivity [86]. 

On the other hand, systemic low-grade inflammation can determine stem cell aging through the activation of the signaling pathways (NF-κB, TOR, JAK/STAT) [87]. Senescent cells can have a negative effect on NF-κB activity only in cells actively involved in inflammation [88]. This is possible due to the ability of senescent cells to express two microRNAs (mir-146a, mir-146b) [89]. All of these take part in the secretion of an increased amount of proinflammatory cytokines, which, in turn, affects the differentiation and function of stem cells, causing, in the end, their aging [90]. 

The number of the senescent cells increases during lifetime. This numerical growth is possible through two mechanisms: either production is faster than disposal, or there is an incomplete clearance [91,92]. The incidence of senescent cells in aged human bodies is estimated to be between 1% to 15%, depending on the studied tissue [89]. A direct link between senescent cells and age-related systemic diseases [atherosclerosis, osteoarthritis] has been demonstrated by in vivo studies that have revealed these cells in the affected tissues [32,93].

An important role in the process of replicative senescence and age-related diseases belongs to DNA damage response, which is directly related to telomere shortening [92]. DNA damage response favors proinflammatory status through its action on stem cells, fibroblasts, or macrophages, thus exacerbating the phenomenon of inflammaging [94,95]. 

**Figure 9:**
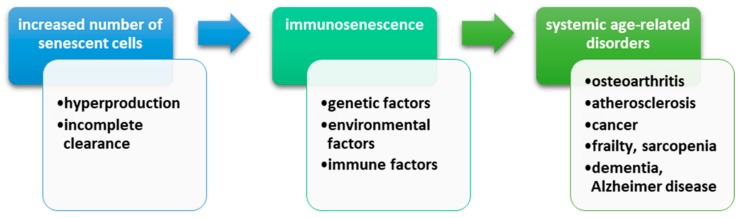
The mechanism of age-related disorders. Senescent cells have an important role in aging through the secretion of matrix-degrading proteins and proinflammatory cytokines, which is called “senescence-associated secretory phenotype”. The number of senescent cells increases during lifetime. This numerical growth is possible through two mechanisms: either production is faster than disposal, or there is an incomplete clearance. Furthermore, senescent cells, though a precise pathway that involves the release of certain mediators and the stop of proliferative activity, can determine their clearing and tissue regeneration. This process is affected in old tissues, leading to the accumulation of these senescent cells. A direct link between senescent cells and age-related systemic diseases (atherosclerosis, osteoarthritis, cancer, sarcopenia, Alzheimer’s disease) has been demonstrated by in vivo studies that have revealed these cells in the affected tissues.

Figure 9 schematically illustrates all the mechanisms that determine the phenomenon of immunosenescence leading to various systemic disorders strictly related to aging process of the organism. 

Aging is associated with structural and functional changes both in the articular cartilage and other anatomical structures such as the synovial membrane, the subchondral bone, or the periarticular tissues (muscles, ligaments, tendons) [96]. The articular cartilage gradually loses its secretory and proliferative capacity but maintains its ability to secrete matrix degrading enzymes and proinflammatory cytokines, thus achieving a senescent secretory phenotype [97]. Moreover, studies have highlighted senescence-associated β-galactosidase (SA-β-gal) staining only in OA cartilage [32]. 

Senescence-associated secretory phenotype participates in maintaining local and systemic low-grade inflammation and is made of chemokines, growth factors, proinflammatory cytokines, and matrix metalloproteinases [29]. The vast majority of senescence-associated secretory phenotype components were found in synovial fluid or in synovial membrane in patients with OA [97,98,99].

An important role in the development of cellular senescence is assigned to DNA damage and activation of p38 MAP kinase [100]. In OA cartilage, studies have demonstrated the presence of chondrocytes sharing DNA damage, as well as an increased expression of p16 INK4a (cyclin-dependent kinase inhibitor) [34,101]. The increased expression of p16 INK4a then activates pRB tumor suppressor, a mechanism by which cell proliferation is regulated through the formation of senescent-associated heterochromatin foci [102].

### 3.4. Autophagy

Autophagy is a cellular mechanism which maintains normal function and homeostasis of the cells through removal of abnormal substances via lysomal degradation [103]. This process stops inflammasome accumulation, thereby reducing systemic inflammation and increasing longevity. 

In the aging process there is a decrease in the autophagy capacity which determines pronounced proinflammatory responses and mitochondrial damage [10]. Lysosome damage increases oxidative stress by increasing the reactive oxygen species, which activates the inflammatory cascade characterized especially by high levels of proinflammatory cytokines such as IL-18 or IL-1β [10]. 

Autophagy defects are associated in the elderly with accumulation of adipose tissue around and within the organs. Many of the adipokines play a proinflammatory role. Leptin, a highly studied adipokine, has endocrine and paracrine roles [104]. Leptin plays an important role in inflammation by stimulating the production of proinflammatory cytokines, by activating natural killer lymphocytes, or by activating monocytes and transforming them into macrophages [105].

**Figure 10:**
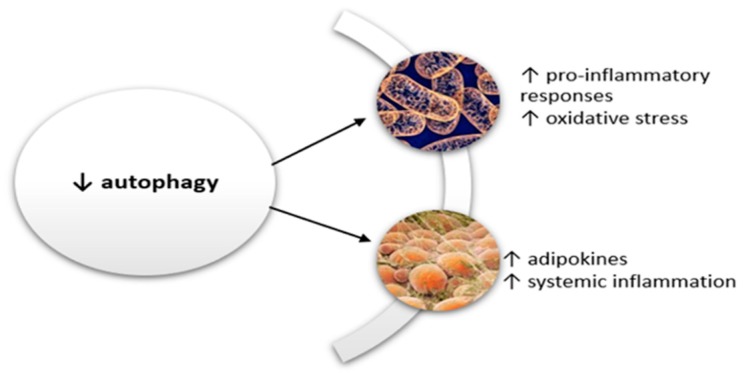
Autophagy, aging process, and systemic low-grade inflammation. In the aging process there is a decrease in the autophagy capacity, which determines pronounced proinflammatory responses and mitochondrial damage. The decrease in autophagy contributes to the inflammation, especially through direct participation in the formation of the proinflammatory state. It stimulates the oxidative stress by mitochondrial damage and favors the formation of adipokines with an important role in inflammation. Lysosomal damage increases the oxidative stress by increasing the reactive oxygen species, which activates the inflammatory cascade characterized especially by high levels of proinflammatory cytokines such as IL-18 or IL-1β. Furthermore, autophagy defects are associated with accumulation of adipose tissue. Many of these adipokines own a proinflammatory role.

The decrease in autophagy contributes to the inflammation, especially through direct participation in the formation of the proinflammatory state. On the one hand, it stimulates the oxidative stress by mitochondrial damage and, on the other hand, favors the formation of adipokines with an important role in inflammation. A vicious circle is formed, in the center of which there is low grade systemic inflammation, the pillar of subsequent changes associated with various systemic disorders (Figure 10).

Autophagy is an important cellular homeostatic mechanism implied in the removal of altered or dysfunctional organelles and macromolecules, being increased by different types of stresses [106,107]. In chondrocytes and cartilage affected by OA, autophagy processes are at high levels in order to regulate changes in OA-like gene expression by modulation of apoptosis and reactive oxygen species, especially during the initial degenerative phase [108]. Autophagy has a cytoprotective effect for articular cartilage, osteoarthritis being associated with a decreased autophagy [107], which in turn leads to the accumulation of destructive macromolecules and determines the loss of the extracellular matrix (ECM), cell dysfunction, and death. Animal studies have indicated that the activation of autophagy can prevent cartilage from mechanical damage in OA [109]. Because it has been demonstrated that mTOR decreases autophagy targeting the mTOR signaling pathway results in increased autophagy signalling and secondary reduces apoptosis, articular cartilage degradation, and synovial fibrosis thus protecting from osteoarthritis [110].

### 3.5. Cellular Apoptosis 

Apoptosis represents a programmed cell death [111], which has an important role in many chronic disorders including OA. The link between apoptosis and arthritis was highlighted in a study that showed the phagocytosis of aged neutrophils by macrophages [112]. Since this study, many others have shown an increase in apoptosis, especially in patients with rheumatoid arthritis [113,114].

Moreover, when apoptosis is affected it leads to the accumulation of dysfunctional cells, reducing the immunological space and thus promoting cancers or infections, which could otherwise be reduced through the correct modulation of the immune system [4].

With aging, studies showed a decrease in the number of chondrocytes in the articular cartilage, this chondrocyte death being closely related to accelerated apoptosis [114]. Data proved that in the case of transgenic mice lacking collagen type II there is an increase in the phenomenon of apoptosis [115]. Other authors have stated that there is an anatomical association between extracellular matrix depletion and cellular apoptosis, and these are related to mechanical stress at the joint [116].

Studies are talking about an “unusual apoptosis” in chondrocytes because these cells suffer morphological and structural changes such as nucleus fragmentation or chromatin condensation [117]. This particular apoptosis of chondrocytes carries the name of “chondroptosis” in the literature. The main characteristics of chondroptosis are increased number of primary lysosomes and Golgi systems, the presence of autophagic vacuoles, and many cellular components in lacunae and extracellular matrix [118]. 

OA is a degenerative disorder characterized by extracellular matrix lesions such as collagen fiber degradation and decreased proteoglycan synthesis. All this affects the attachment of chondrocytes to the extracellular matrix, which leads to the acceleration of apoptosis [119]. Moreover, in this process, integrin blocking also accelerates cartilage degradation [120]. 

Other factors involved in the occurrence of OA and apoptosis of chondrocytes are the granzyme B that induces apoptosis with the help of perforin, both being released from granules of cytotoxic cells [121]. 

MAC (membrane attack complex) also favors the increase of local inflammation and the activation of degrading enzymes such us MMP [122]. 

Cellular apoptosis is a very well controlled process in the body, having an important role throughout life. Initiation of apoptosis can be made using two pathways. The intrinsic pathway is based on cellular stress and intracellular signals that cause initiation of programmed cell death, while the extrinsic pathway refers to signals received from other cells [123,124]. Both mechanisms eventually trigger caspase activation. 

The intrinsic pathway may be called the mitochondrial pathway, being closely related to mitochondrial proteins from the intermembrane space [125]. Mitochondrial changes can be expressed either by increasing membrane permeability or by membrane pore formation which may lead to mitochondrial swelling [126]. Nitric oxide has an important role in the induction of apoptosis by increasing the mitochondrial membrane permeability, acting on the membrane potential [127].

Mitochondrial proteins (SMACs—second mitochondria-derived activator of caspases) can bind IAPs (inhibitors of apoptosis) leading to their deactivation, activation of caspases, and further apoptosis [128]. Moreover, the MAC (mitochondrial apoptosis-induced channel) releases the cytochrome c that forms the apoptosome binding with Apaf-1 (apoptotic protease activating factor-1) and pro-caspase-9 [129]. Anti-apoptotic genes such as Bcl-2 family that encode numerous proteins can have a direct action on the MAC. Thus, the genes were divided into two categories: pro-apoptosis —BAX, BID, BAK, or BAD and against apoptosis—Bcl-2, Bcl-Xl, or Mcl-1 [130].

The extrinsic mechanism of apoptosis is based on the binding of extracellular ligands with different membrane receptors, which results in DISC (death-inducing signaling complex) formation [125]. This extrinsic mechanism refers to two major cellular pathways: TNF path and Fas path [131]. 

TNFα is considered to be the major extrinsic mediator of apoptosis. The binding between TNFα and TNFR1 causes caspase activation and initiation of apoptosis [132]. On the other hand, Fas ligand (FasL) binds to Fas receptor, causing DISC formation and activation of caspases 8 and 10. In this way, the cellular apoptosis process is initiated [131]. 

Caspases have a central role in apoptosis. They can be divided into two categories: initiator caspases (caspase 2,8,9,10,11,12) and effector caspases (caspase 3,6,7). Activation of these caspases occurs in the cascade and acts through degradative enzymes. Not all apoptotic processes are dependent on caspase activation; there are also AIF (apoptosis-inducing factor)-controlled pathways [133].

## 4. Conclusions

Aging is an inevitable process in the human body which is associated with a multitude of systemic and localized changes. All these conditions have a common pathogenic mechanism characterized by the presence of a low-grade proinflammatory status. As we have previously seen, inflammaging has a multifactorial aetiology including an increased number of proinflammatory cytokines, oxidative stress, immunosenescence, autophagy, or cellular DNA damage. The incidence of OA is steadily increasing, especially among the elderly. The mechanism of articular cartilage degeneration is not necessarily the consequence of aging, but aging is considered to be a risk factor for the occurrence of OA. There is a close relationship between chondrocyte activity and local articular environment changes due to cell senescence followed by secretion of inflammatory mediators. Furthermore, systemic inflammaging can lead to cartilage destruction, pain, disability, and an impaired quality of life. Future research and therapeutic perspectives with respect to longevity should address the pathophysiological mechanisms underlying inflammaging as well as therapy to improve the capacity of the human organism to adapt to damaging agents that increase with age.

## Figures and Tables

**Table 1:** Characteristics of chondrosenescence.

Senescent Chondrocytes	References
formation of senescence-associated heterochromatin	[30]
**Increase of:**	
expression of cyclin-dependent kinase inhibitor (p16INK4a)	[30,31,32]
expression of senescence-associated β-galactosidase	[30,32]
telomere length reduction	[34]
free oxygen radicals, ↑ oxidative stress	[35]
Matrix Metallopeptidase 13 (MMP-13)	[28,36]
**Decrease of:**	
transforming growth factor-beta (TGF-β)	[37]
platelet derived growth factor (PDGF)	[37]
insulin-like growth factor-I (IGF-I)	[37]
synthesis of proteoglycans	[38,39]

**Table 2:** Oxidative stress, aging and osteoarthritis.

Increase in Oxidative Stress Leads to	
Acceleratedcatabolic Processes with Increase in	Slowed Anabolic Processes with Decrease in
proinflammatory cytokines	extracellular matrix
MMP	growth factor expression
accumulation of cellular detrudes	protein synthesis
apoptosis	DNA synthesis

## Abbreviations

ROS	Reactive oxygen species
TNF α	Tumor necrosis factor alpha
IFN γ	Interferon gamma
IL	Interleukins
CRP	C reactive protein
DNA	Dezoxiribonucleic acid
ATP	Adenosine triphosphate
RNA	Ribonucleic acid
OA	Osteoarthritis
MMP	Matrix metalloproteinase
PDGF	Platelet derived growth factor
TGF-β	Transforming growth factor beta
IGF-I	Insulin-like growth factor-I
BMP-6	Morphogenic protein 6
p16INK4a	Cyclic-dependent kinase inhibitor
MAC	Membrane attack complex
SMACs	Second mitochondria-derived activator of caspases
IAPs	Inhibitors of apoptosis
Apaf-1	Apoptotic protease activating factor-1
DISC	Death inducing signaling complex
FasL	Fas ligand

## References

[1] Bucci L., Ostan R., Capri S., Salvioli S., Cevenini E., Celani L., Monti D., Franceschi C., Fulop T., Franceschi C., Hirokawa K., Pawelec G. (2009). Inflamm-Aging: Handbook on Immunosenesce—basic Understanding and Clinical Applications 1.

[2] Rezus E., Floria M., Grigoriu A., Tamba B., Rezus C. (2015). Cardiovascular Risk Factors in Chronic Inflammatory Rheumatic Diseases: Modern Assessment and Diagnosis. Curr. Vasc. Pharmacol..

[3] Franceschi C., Bonafe M., Valensin S., Olivieri F., De Luca M., Ottaviani E., De Benedictis G. (2000). Inflamm-aging: An evolutionary perspective on immunosenescence. Ann. N. Y. Acad. Sci..

[4] Ventura M.T., Casciaro M., Gamgemi S., Buquicchio R. (2017). Immunosenescence in aging: Between immune cells depletion and cytokines up-regulation. Clin. Mol. Allergy.

[5] Candela M.E., Yasuhara R., Iwamoto M., Enomoto-Iwamoto M. (2014). Resident mesenchymal progenitors of articular cartilage. Matrix Biol..

[6] Ashraf S., Cha B.H., Kim J.S., Ahn J., Han I., Park H., Lee S.H. (2016). Regulation of senescence associated signaling mechanisms in chondrocytes for cartilage tissue regeneration. Osteoarthr. Cartil..

[7] Brandl A., Hartmann A., Bechmann V., Graf B., Nerlich M., Angele P. (2011). Oxidative stress induces senescence in chondrocytes. J. Orthop. Res..

[8] Dillon C.F., Rasch E.K., Gu Q., Hirsch R. (2006). Prevalence of knee osteoarthritis in the United States: Arthritis data from the Third National Health and Nutrition Examination Survey. J. Rheumatol..

[9] Mobasheri A., Matta C., Zákány R., Musumeci G. (2015). Chondrosenescence: Definition, hallmarks and potential role in the pathogenesis of osteoarthritis. Maturitas.

[10] Salminen A., Kaarniranta K., Kauppinen A. (2012). Inflammaging: Disturbed interplay between autophagy and inflammasomes. Aging.

[11] Findlay D.M. (2007). Vascular pathology and osteoarthritis. Rheumatology.

[12] Altindag O., Erel O., Aksoy N., Selek S., Celik H., Karaoglanoglu M. (2007). Increased oxidative stress and its relation with collagen metabolism in knee osteoarthritis. Rheumatol. Int..

[13] Lepetsos P., Papavassiliou A.G. (2016). ROS/oxidative stress signaling in osteoarthritis. Biochim. Biophys. Acta (BBA).

[14] Yudoh K., van Trieu N., Nakamura H., Hongo-Masuko K., Kato T., Nishioka K. (2005). Potential involvement of oxidative stress in cartilage senescence and development of osteoarthritis: Oxidative stress induces chondrocyte telomere instability and down regulation of chondrocyte function. Arthritis Res. Ther..

[15] Goldring S.R., Goldring M.B. (2016). Changes in the osteochondral unit during osteoarthritis: Structure, function and cartilage–bone crosstalk. Nat. Rev. Rheumatol..

[16] Ershler W.B. (1993). Interleukin-6: A cytokine for gerontologists. J. Am. Geriatr. Soc..

[17] Bild V., Ababei D.C., Neamtu M., Vasincu A., Bild W., Stanciu G.D., Tamba B.I., Solcan G., Beschea Chiriac S. (2017). Isobolar analysis of the binary fixed-ratio combination of acetylsalicilic acid-acetaminophen. Farmacia.

[18] Bruunsgaard H. (2002). Effects of tumor necrosis factor-alpha and interleukin-6 in elderly populations. Eur. Cytokine Netw..

[19] Spector T.D., Hart D.J., Nandra D., Doyle  D.V., Mackillop N., Gallimore J.R., Pepys M.B. (1997). Low-level increases in serum C-reactive protein are present in early osteoarthritis of the knee and predict progressive disease. Arthritis Rheum..

[20] Livshits G., Zhai G., Hart D.J., Kato B.S., Wang H., Williams F.M., Spector T.D. (2009). Interleukin-6 is a significant predictor of radiographic knee osteoarthritis: The Chingford study. Arthritis Rheum..

[21] Penninx B.W., Abbas H., Ambrosius W., Nicklas B.J., Davis C., Messier S.P., Pahor M. (2004). Inflammatory markers and physical function among older adults with knee osteoarthritis. J. Rheumatol..

[22] Iurea (Raţă) D.M., Popa M., Chailan J.F., Tamba B.I., Tudorancea I., Peptu C.A. (2013). Ibuprofen-loaded chitosan/poly (maleic anhydride-alt-vinyl acetate) submicronic capsules for pain treatment. J. Bioact. Compat. Polym..

[23] Alexa T., Marza A., Voloseniuc T., Tamba B. (2015). Enhanced analgesic effects of tramadol and common trace element coadministration in mice: Coadministration of Tramadol and Trace Elements in Mice. J. Neurosci. Res..

[24] Uritu C.M., Mihai C.T., Stanciu G.D., Dodi G., Alexa-Stratulat T., Luca A., Leon-Constantin M.M., Stefanescu R., Bild V., Melnic S. (2018). Medicinal plants of the family Lamiaceae in pain therapy: A review. Pain Res. Manag..

[25] Tamba B.I., Petreus T., Constantin M.M.L., Rezus C., Floria M., Rezus E. (2015). Heavy metal trace elements induced antinociception in an experimental mouse model. Rev. Chim..

[26] Stannus O.P., Jones G., Blizzard L., Cicuttini F.M., Ding C. (2013). Associations between serum levels of inflammatory markers and change in knee pain over 5 years in older adults: A prospective cohort study. Ann. Rheum. Dis..

[27] Long D., Blake S., Song X.Y., Lark M., Loeser R.F. (2008). Human articular chondrocytes produce IL-7 and respond to IL-7 with increased production of matrix metalloproteinase-13. Arthritis Res. Ther..

[28] Forsyth C.B., Cole A., Murphy G., Bienias J.L., Im H.J., Loeser R.F. (2005). Increased matrix metalloproteinase-13 production with aging by human articular chondrocytes in response to catabolic stimuli. J. Gerontol. Ser. A Biol. Sci. Med. Sci..

[29] Coppe J.P., Patil C.K., Rodier F., Sun Y., Munoz D.P., Goldstein J., Nelson P.S., Desprez P.Y., Campisi J. (2008). Senescence-associated secretory phenotypes reveal cellnonautonomous functions of oncogenic RAS and the p53 tumor suppressor. PLoS Biol..

[30] Wang Q., Rozelle A.L., Lepus C.M., Scanzello C.R., Song J.J., Larsen D.M., Crish J.F., Bebek G., Ritter S.Y., Lindstrom T.M. (2011). Identification of a central role for complement in osteoarthritis. Nat. Med..

[31] Campisi J. (2011). Cellular senescence: Putting the paradoxes in perspective. Curr. Opin. Genet. Dev..

[32] Price J.S., Waters J.G., Darrah C., Clark I.M. (2002). The role of chondrocyte senescence in osteoarthritis. Aging Cell.

[33] Haulica I., Bild W., Mihaila C., Serban D.N., Serban L., Boisteanu D., Ionita T., Radasanu O. (2004). Comparative study of the inhibitory effects of adrenomedullin on angiotensin II contraction in rat conductance and resistance arteries. J. Renin. Angiotensin Aldosterone Syst..

[34] Zhou H.W., Lou S.Q., Zhang K. (2004). Recovery of function in osteoarthritic chondrocytes induced by p16INK4a-specific siRNA in vitro. Rheumatology.

[35] Zhai G., Aviv A., Hunter D.J., Hart D.J., Gardner J.P., Kimura M., Lu X., Valdes A.M., Spector T.D. (2006). Reduction of leucocyte telomere length in radiographic hand osteoarthritis: A population-based study. Ann. Rheum. Dis..

[36] Kurz B., Lemke A.K., Fay J., Pufe T., Grodzinsky A.J., Schünke M. (2005). Pathomechanisms of cartilage destruction by mechanical injury. Ann. Anat..

[37] Mouritzen U., Christgau S., Lehmann H.J., Tanko L.B., Christiansen C. (2003). Cartilage turnover assessed with a newly developed assay measuring collagen type II degradation products: Influence of age, sex, menopause, hormone replacement therapy, and body mass index. Ann. Rheum. Dis..

[38] Guerne P.A., Blanco F., Kaelin A., Desgeorges A., Lotz M. (1995). Growth factor responsiveness of human articular chondrocytes in aging and development. Arthritis Rheumol..

[39] Bobacz K., Gruber R., Soleiman A., Erlacher L., Smolen J.S., Graninger W.B. (2003). Expression of bone morphogenetic protein 6 in healthy and osteoarthritic human articular chondrocytes and stimulation of matrix synthesis in vitro. Arthritis Rheum..

[40] Yabluchanskiy A., Ungvari Z., Csiszar A., Tarantini S. (2018). Advances and challenges in geroscience research: An update. Physiol. Int..

[41] Morrisette-Thomas V., Cohen A.A., Fulop T., Riesco E., Legault V., Li Q., Milot E., Dusseault-Bélanger F., Ferrucci L. (2014). Inflamm-aging does not simply reflect increases in pro-inflammatory markers. Mech. Ageing Dev..

[42] Singh T., Newman A.B. (2011). Inflammatory markers in population studies of aging. Ageing Res. Rev..

[43] Lopez-Otin C., Blasco M.A., Partridge L., Serrano M., Kroemer G. (2013). The hallmarks of aging. Cell.

[44] Mobasheri A., Vannucci S.J., Bondy C.A., Carter S.D., Innes J.F., Arteaga M.F., Trujillo E., Ferraz I., Shakibaei M., Martín-Vasallo P. (2002). Glucose transport and metabolismin chondrocytes: A key to understanding chondrogenesis, skeletal development and cartilage degradation in osteoarthritis. Histol. Histopathol..

[45] Salvioli S., Capri M., Valensin S., Tieri P., Monti D., Ottaviani E., Franceschi C. (2006). Inflamm-aging, cytokines and aging: State of the art, new hypotheses on the role of mitochondria and new perspectives from systems biology. Curr. Pharm. Des..

[46] Maggio M., Guralnik J.M., Longo D.L., Ferrucci L. (2006). Interleukin-6 in aging and chronic disease: A magnificent pathway. J. Gerontol. Ser. A Biol. Sci. Med. Sci..

[47] De Martinis M., Franceschi C., Monti D., Ginaldi L. (2005). Inflamm-ageing and lifelong antigenic load as major determinants of ageing rate and longevity. FEBS Lett..

[48] Di Bona D., Vasto S., Capurso C., Christiansen L., Deiana L., Franceschi C., Hurme M., Mocchegiani E., Rea M., Lio D. (2009). Effect of interleukin-6 polymorphisms on human longevity: A systematic review and meta-analysis. Ageing Res. Rev..

[49] Lio D., Scola L., Crivello A., Colonna-Romano G., Candore G., Bonafé M., Cavallone L., Marchegiani F., Olivieri F., Franceschi C. (2003). Inflammation, genetics, and longevity: Further studies on the protective effects in men of IL-10 -1082 promoter SNP and its interaction with TNF-alpha-308 promoter SNP. J. Med. Genet..

[50] Adams A.A., Breathnach C.C., Katepalli M.P., Kohler K., Horohov D.W. (2008). Advanced age in horses affects divisional history of T cells and inflammatory cytokine production. Mech. Ageing Dev..

[51] Evans W.J., Paolisso G., Abbatecola A.M., Corsonello A., Bustacchini S., Strollo F., Lattanzio F. (2010). Frailty and muscle metabolism dysregulation in the elderly. Biogerontology.

[52] Rea I.M., Ross O.A., Armstrong M., McNerlan S., Alexander D.H., Curran M.D., Middleton D. (2003). Interleukin-6-gene C/G 174 polymorphism in nonagenarian and octogenarian subjects in the BELFAST study. Reciprocal effects on IL-6, soluble IL-6 receptor and for IL-10 in serum and monocyte supernatants. Mech. Ageing Dev..

[53] Lio D., Candore G., Crivello A., Scola L., Colonna-Romano G., Cavallone L., Hoffmann  E., Caruso M., Licastro F., Caldarera C.M. (2004). Opposite effects of interleukin 10 common gene polymorphisms in cardiovascular diseases and in successful ageing: Genetic background of male centenarians is protective against coronary heart disease. J. Med. Genet..

[54] Pes G.M., Lio D., Carru C., Deiana L., Baggio G., Franceschi C., Ferrucci L., Oliveri F., Scola L., Crivello A. (2004). Association between longevity and cytokine gene polymorphisms. A study in Sardinian centenarians. Aging Clin. Exp. Res..

[55] O’Donovan A., Pantell M.S., Puterman E., Dhabhar F.S., Blackburn E.H., Yaffe K., Cawthon R.M., Opresko P.L., Hsueh W.C., Satterfield S. (2011). Health Aging and Body Composition Study: Cumulative inflammatory load is associated with short leukocyte telomere length in the Health, Aging and Body Composition Study. PLoS ONE.

[56] Shiels P.G.S., McGlynn L.M., MacIntyre A., Johnson P.C., Batty G.D., Burns H., Cavanagh J., Deans K.A., Ford I., McConnachie A. (2011). Accelerated telomere attrition is associated with relative household income, diet and inflammation in the pSoBid cohort. PLoS ONE.

[57] Schett G., Elewaut D., McInnes I.B., Dayer J.-M., Neurath M.F. (2013). How cytokine networks fuel inflammation: Toward a cytokine-based disease taxonomy. Nat. Med..

[58] Bastiaansen-Jenniskens Y., Saris D., Creemers L.B., Grässel S., Aszódi A. (2017). Pro- and Anti-inflammatory Cytokine Profiles in Osteoarthritis. Cartilage.

[59] Bai Y., Gao S., Liu Y., Jin S., Zhang H., Su K. (2019). Correlation between Interleukin-17 gene polymorphism and osteoarthritis susceptibility in Han Chinese population. BMC Med. Genet..

[60] Vicenti G., Bizzoca D., Carrozzo M., Solarino G., Moretti B. (2018). Multi-omics analysis of synovial fluid: A promising approach in the study of osteoarthritis. J. Biol. Regul. Homeost. Agents.

[61] Ding L., Hong X., Sun B., Huang Q., Wang X., Liu X., Li L., Huang Z., Liu D. (2017). IL-37 is associated with osteoarthritis disease activity and suppresses proinflammatory cytokines production in synovial cells. Sci. Rep..

[62] Cannizzo E.S., Clement C.C., Sahu R., Follo C., Santambrogio L. (2011). Oxidative stress, inflammaging and immunosenescence. J. Proteom..

[63] Alexa A.I., Cantemir A., Antioch I., Balmus I.M., Cojocaru S., Luca A., Filip M.A., Ababei C.D., Zamfir C.L. (2017). The Dynamics of the Main Oxidative Stress Chemical Markers in the Serum of Rats Stressed by Various Behavioural Tasks. Rev. Chim..

[64] Ginaldi L., De Martinis M., Monti D., Franceschi C. (2004). The immune system in the elderly: Activation-induced and damage-induced apoptosis. Immunol. Res..

[65] Marchal J., Pifferi F., Aujard F. (2013). Resveratrol in mammals: Effects on aging biomarkers, age-related diseases, and life span. Ann. N. Y. Acad. Sci..

[66] Draganescu D., Ibanescu C., Tamba B.I., Andritoiu C.V., Dodi G., Popa M.I. (2015). Flaxseed lignan wound healing formulation: Characterization and in vivo therapeutic evaluation. Int. J. Biol. Macromol..

[67] De la Fuente M., Miquel J. (2009). An update of the oxidation-inflammation theory of aging: The involvement of the immune system in oxi-inflamm-aging. Curr. Pharm. Des..

[68] Dorn G.W. (2013). Molecular mechanisms that differentiate apoptosis from programmed necrosis. Toxicol. Pathol..

[69] Freund A., Orjalo A.V., Desprez P.Y., Campisi J. (2010). Inflammatory networks during cellular senescence: Causes and consequences. Trends Mol. Med..

[70] Imhof H., Sulzbacher I., Grampp S., Czerny C., Youssefzadeh S., Kainberger F. (2000). Subchondral bone and cartilage disease: A rediscovered functional unit. Investig. Radiol..

[71] Hiran T.S., Moulton P.J., Hancock J.T. (1998). In situ detection of superoxide anions within porcine articular cartilage. Br. J. Biomed. Sci..

[72] Hiran T.S., Moulton P.J., Hancock J.T. (1997). Detection of superoxide and NADPH oxidase in porcine articular chondrocytes. Free Radic. Biol. Med..

[73] Clancy R.M., Amin A.R., Abramson S.B. (1998). The role of nitric oxide in inflammation and immunity. Arthritis Rheum..

[74] Casagrande D., Stains J.P., Murthi A.M. (2015). Identification of shoulder osteoarthritis biomarkers: Comparison between shoulders with and without osteoarthritis. J. Shoulder Elbow Surg..

[75] Balaganur V., Pathak N.N., Lingaraju M.C., More A.S., Latief N., Kumari R.R., Kumar D., Tandan S.K. (2014). Effect of Smethylisothiourea, an inducible nitric oxide synthase inhibitor, in joint pain and pathology in surgically induced model of osteoarthritis. Connect. Tissue Res..

[76] Ramage L., Martel M.-A., Hardingham G.E., Salter D.M. (2008). NMDA receptor expression and activity in osteoarthritic human articular chondrocytes. Osteoarthr. Cartil..

[77] Gokay N.S., Yilmaz I., Komur B., Demiroz A.S., Gokce A., Devisoglu S., Gokay B.V. (2016). A Comparison of the effects of neuronal nitric oxide synthase and inducible nitric oxide synthase inhibition on cartilage damage. BioMed Res. Int..

[78] Leonidou A., Lepetsos P., Mintzas M., Kenanidis E., Macheras G., Tzetis M., Potoupnis M., Tsiridis E. (2018). Inducible nitric oxide synthase as a target for osteoarthritis treatment. Expert Opin. Ther. Targets.

[79] Alderton W.K., Cooper C.E., Knowles R.G. (2001). Nitric oxide synthases: Structure, function and inhibition. Biochem. J..

[80] Studer R., Jaffurs D., Stefanovic-Racic M., Robbins P.D., Evans C.H. (1999). Nitric oxide in osteoarthritis. Osteoarthr. Cartil..

[81] Stanciu G.D., Solcan G. (2016). Acute idiopathic polyradiculoneuritis concurrent with acquired myasthenia gravis in a West Highland white terrier dog. BMC Vet. Res..

[82] Demaria M., Ohtani N., Youssef S.A., Rodier F., Toussaint W., Mitchell J.R., Laberge R.M., Vijg J., Van Steeg H., Dollé M.E. (2014). An essential role for senescent cells in optimal wound healing through secretion of PDGF-AA. Dev. Cell.

[83] Mosteiro L., Pantoja C., Alcazar N., Marión R.M., Chondronasiou D., Rovira M., Fernandez-Marcos P.J., Muñoz-Martin M., Blanco-Aparicio C., Pastor J. (2016). Tissue damage and senescence provide critical signals for cellular reprogramming in vivo. Science.

[84] Muñoz-Espín D., Serrano M. (2014). Cellular senescence: From physiology to pathology. Nat. Rev. Mol. Cell Biol..

[85] Balistreri C.R., Candore G., Accardi G., Colonna-Romano G., Lio D. (2013). NF-κB pathway activators as potential ageing biomarkers: Targets for new therapeutic strategies. Immun. Ageing.

[86] Weiskopf D., Weinberger B., Grubeck-Loebenstein B. (2009). The aging of the immune system. Transpl. Int..

[87] Bruunsgaard H., Pedersen M., Pedersen B.K. (2001). Aging and proinflammatory cytokines. Curr. Opin. Hematol..

[88] Taganov K.D., Boldin M.P., Chang K.J., Baltimore D. (2006). NF-kappaB dependent induction of microRNA miR-146, an inhibitor targeted to signaling proteins of innate immune responses. Proc. Natl. Acad. Sci. USA.

[89] Bhaumik D., Scott G.K., Schokrpur S., Patil C.K., Orjalo A.V., Rodier F., Lithgow G.J., Campisi J. (2009). MicroRNAs miR-146a/b negatively modulates the senescence-associated inflammatory mediators IL-6 and IL-8. Aging.

[90] Jones D.L., Rando T.A. (2011). Emerging models and paradigms for stem cell ageing. Nat. Cell Biol..

[91] Cevenini E., Monti D., Franceschi C. (2013). Inflamm-ageing. Curr. Opin. Clin. Nutr. Metab. Care.

[92] Campisi J., d’Adda di Fagagna F. (2007). Cellular senescence: When bad things happen to good cells. Nat. Rev. Mol. Cell Biol..

[93] Vasile E., Tomita Y., Brown L.F., Kocher O., Dvorak H.F. (2001). Differential expression of thymosin beta-10 by early passage and senescent vascular endothelium is modulated by VPF/VEGF: Evidence for senescent endothelial cells in vivo at sites of atherosclerosis. FASEB J..

[94] Olivieri F., Albertini M.C., Orciani M., Ceka A., Cricca M., Procopio A.D., Bonafè M. (2015). DNA damage response (DDR) and senescence: Shuttled inflamma-miRNAs on the stage of inflamm-aging. Oncotarget.

[95] Bonafè M., Storci G., Franceschi C. (2012). Inflamm-aging of the stem cell niche: Breast cancer as a paradigmatic example: Breakdown of the multi-shell cytokine network fuels cancer in aged people. Bioessays.

[96] Van der Kraan P.M., van den Berg W.B. (2008). Osteoarthritis in the context of ageing and evolution. Loss of chondrocyte differentiation block during ageing. Ageing Res. Rev..

[97] Loeser R.F., Goldring S.R., Scanzello C.R., Goldring M.B. (2012). Osteoarthritis: A disease of the joint as an organ. Arthritis Rheum..

[98] Sohn D.H., Sokolove J., Sharpe O., Erhart J.C., Chandra P.E., Lahey L.J., Erhart J.C., Chandra P.E., Lahey L.J., Lindstrom T.M. (2012). Plasma proteins present in osteoarthritic synovial fluid can stimulate cytokine production via Toll-like receptor 4. Arthritis Res. Ther..

[99] Clutterbuck A.L., Smith J.R., Allaway D., Harris P., Liddell S., Mobasheri A. (2011). High throughput proteomic analysis of the secretome in an explant model of articular cartilage inflammation. J. Proteom..

[100] Freund A., Patil C.K., Campisi J. (2011). p38MAPK is a novel DNA damage response-independent regulator of the senescence-associated secretory phenotype. EMBO J..

[101] Rose J., Soder S., Skhirtladze C., Schmitz N., Gebhard P.M., SesselmAnn S., Aigner T. (2012). DNA damage, discoordinated gene expression and cellular senescence in osteoarthritic chondrocytes. Osteoarthr. Cartil..

[102] Rodier F., Campisi J. (2011). Four faces of cellular senescence. J. Cell Biol..

[103] White E., Lowe S.W. (2009). Eating to exit: Autophagy-enabled senescence revealed. Genes Dev..

[104] Trayhurn P., Drevon C.A., Eckel J. (2011). Secreted proteins from adipose tissue and skeletal muscle—Adipokines, myokines and adipose/muscle cross-talk. Arch. Physiol. Biochem..

[105] De Heredia F.P., Sonia G.M., Ascension M. (2012). Chronic and degenerative diseases: Obesity, inflammation and the immune system. Proc. Nutr. Soc..

[106] Mizushima N., Komatsu M. (2011). Autophagy: Renovation of Cells and Tissues. Cell.

[107] Jeon H., Im G.-I. (2017). Autophagy in osteoarthritis. Connect. Tissue Res..

[108] Li Y.-S., Zhang F.-J., Zeng C., Luo W., Xiao W.-F., Gao S.-G., Lei G.-H. (2016). Autophagy in osteoarthritis. Jt. Bone Spine.

[109] Gao T., Guo W., Chen M., Huang J., Yuan Z., Zhang Y., Wang M., Li P., Peng J., Wang A. (2016). Extracellular Vesicles and Autophagy in Osteoarthritis. BioMed Res. Int..

[110] Zhang Y., Vasheghani F., Li Y., Blati M., Simeone K., Fahmi H., Lussier B., Roughley P., Lagares D., Pelletier J.-P. (2015). Cartilage-specific deletion of mTOR upregulates autophagy and protects mice from osteoarthritis. Ann. Rheum. Dis..

[111] Kerr J.F., Wyllie A.H., Currie A.R. (1972). Apoptosis: A basic biological phenomenon with wide-ranging implications in tissue kinetics. Br. J. Cancer.

[112] Savill J.S., Wyllie A.H., Henson J.E., Savill J.S., Wyllie A.H., Henson J.E., Walport M.J., Henson P.M., Haslett C. (1989). Macrophage phagocytosis of aging neutrophils in inflammation. Programmed cell death in the neutrophil leads to its recognition by macrophages. J. Clin. Investig..

[113] Firestein G.S., Nguyen K., Aupperle K.R., Yeo M., Boyle D.L., Zvaifler N.J. (1996). Apoptosis in rheumatoid arthritis: p53 overexpression in rheumatoid arthritis synovium. Am. J. Pathol..

[114] Hasunuma T., Kayagaki N., Asahara H., Motokawa S., Kobata T., Yagita H., Aono H., Sumida T., Okumura K., Nishioka K. (1997). Accumulation of soluble Fas in inflamed joints of patients with rheumatoid arthritis. Arthritis Rheum..

[115] Zamli Z., Sharif M. (2011). Chondrocyte apoptosis: A cause or consequence of osteoarthritis. Int J. Rheum. Dis..

[116] Yang C., Li S.W., Helminen H.J., Khillan J.S., Bao Y., Prockop D.J. (1997). Apoptosis of chondrocytes in transgenic mice lacking collagen II. Exp. Cell Res..

[117] Hashimoto S., Ochs R.L., Komiya S., Lotz M. (1998). Linkage of chondrocyte apoptosis and cartilage degradation in human osteoarthritis. Arthritis Rheum..

[118] Roach H.I., Aigner T., Kouri J.B. (2004). Chondroptosis: A variant of apoptotic cell death in chondrocytes. Apoptosis.

[119] Aigner T., Kim H.A., Roach H.I. (2004). Apoptosis in osteoarthritis. Rheum. Dis. Clin. N. Am..

[120] Kim H.A., Suh D.I., Song Y.W. (2001). Relationship between chondrocyte apoptosis and matrix depletion in human articular cartilage. J. Rheumatol..

[121] Pulai J.I., del Carlo M., Loeser R.F. (2002). The α5β1 integrin provides matrix survival signals for normal and osteoarthritic human articular chondrocytes in vitro. Arthritis Rheum..

[122] Saito S., Murakoshi K., Kotake S., Kamatani N., Tomatsu T. (2008). Granzyme B induces apoptosis of chondrocytes with natural killer cell-like cytotoxicity in rheumatoid arthritis. J. Rheumatol..

[123] Green D. (2011). Means to an End: Apoptosis and other Cell Death Mechanisms.

[124] Raychaudhuri S. (2010). A minimal model of signaling network elucidates cell-to-cell stochastic variability in apoptosis. PLoS ONE.

[125] Alberts B., Johnson A., Lewis J., Raff M., Roberts K., Walter P. (2008). Chapter 18 Apoptosis: Programmed Cell Death Eliminates Unwanted Cells. Molecular Biology of the Cell (Textbook).

[126] Gonzalez D., Bejarano I., Barriga C., Rodriguez A.B., Pariente J.A. (2010). Oxidative stress-induced caspases are regulated in human myeloid HL-60 cells by calcium signal. Curr. Signal. Transduct. Ther..

[127] Brüne B. (2003). Nitric oxide: NO apoptosis or turning it ON?. Cell Death Differ..

[128] Fesik S.W., Shi Y., Structural Biology (2001). Controlling the caspases. Science.

[129] Dejean L.M., Martinez-Caballero S., Kinnally K.W. (2006). Is MAC the knife that cuts cytochrome c from mitochondria during apoptosis?. Cell Death Differ..

[130] Dejean L.M., Martinez-Caballero S., Manon S., Kinnally K.W. (2006). Regulation of the mitochondrial apoptosis-induced channel, MAC, by BCL-2 family proteins. Biochim. Biophys. Acta.

[131] Wajant H. (2002). The Fas signaling pathway: More than a paradigm. Science.

[132] Chen G., Goeddel D.V. (2002). TNF-R1 signaling: A beautiful pathway. Science.

[133] Susin S.A., Lorenzo H.K., Zamzami N., Marzo I., Snow B.E., Brothers G.M., Mangion J., Jacotot E., Costantini P., Loeffler M. (1999). Molecular characterization of mitochondrial apoptosis-inducing factor. Nature.

